# Red background color biases gender categorization of human faces

**DOI:** 10.1038/s41598-023-34644-4

**Published:** 2023-05-13

**Authors:** Na Chen, Koyo Nakamura, Katsumi Watanabe

**Affiliations:** 1 The Gonda Multidisciplinary Brain Research Center, Bar-Ilan University, 5290002 Ramat Gan, Israel; 2 Faculty of Psychology, Department of Cognition, Emotion, Methods in Psychology, University of Vienna, 1010 Vienna, Austria; 3 Japan Society for the Promotion of Science, Tokyo, 102-0083 Japan; 4 Faculty of Science and Engineering, Waseda University, Tokyo, 169-8555 Japan

**Keywords:** Human behaviour, Colour vision, Social behaviour

## Abstract

Color carries gender information (e.g., red-female). This study explored whether background color could influence the gender categorization of human faces. Visual stimuli were generated from faces whose sexually dimorphic content was morphed monotonically from female to male perception. The face stimulus was presented upright (Experiment 1) and inverted (Experiment 2) with three background colors (i.e., red, green, and gray). Participants were instructed to categorize the gender of the face stimulus as male or female by pressing one of two labelled keys. Results showed that a red background could bias the gender of an ambiguous upright face toward a female compared with green and gray background colors (Experiment 1). However, this red effect was diminished when the face stimulus was inverted (Experiment 2). These results suggest that red background color interacting with facial configuration features biases gender perception toward a female face, possibly through top-down processing of learned associations between the color red and femininity.

## Introduction

Colors are associated with gender stereotypes (e.g., pink-girl/blue-boy)^1–5^. In many countries, parents have different color preferences for male versus female children, and children are exposed to colors related to their genders. For instance, girls’ toys, clothes, and rooms are typically colored in variations of pink/reddish colors while boys are surrounded by blue/green colors^2–5^. Thus, mere exposure to gendered colors during early perception may lead to color-gender associations that influence gender-related cognition and behavior.

In Japan, reddish and bluish colors are often used to represent female and male gender symbols, respectively, in social and public service systems. For instance, the symbol of the female lavatory is often colored in red or pink while that of the male lavatory is often colored in blue or green^6–8^. It is common for reddish colors to be widely used in clothes, cosmetics, and activities for girls and women while blue/green colors are more frequently used in male clothes and behaviors/interests in the modern daily life. These gendered-color encounters in environment and social interactions may also lead to the gender difference in color preferences. Both adults^9–12^ and children^13–20^ are reported to have gender difference in color preferences with girls and women preferring reddish colors and boys and men preferring blue/green colors. For instance, Wang and Hines^20^ reported girls at the age around 2 showed a greater preference for the pink toys than boys. Moreover, studies on color connotations showed that pink/reddish colors are considered feminine while blue/green are considered masculine^21,22^. The connotations and preferences associated with color and gender, as well as statistical learning from co-occurrences in social interactions, may provide the basis for the formation of color-gender associations.

Numerous studies have been examined the effect of color on psychological functioning. According to Elliot and colleagues' color-in-context theory, colors convey specific meanings that can be learned from the environment and social interactions, as well as being evolutionarily based. The influence of color is also dependent on psychological contexts^23,24^. For instance, the influence of the color red on approach or avoidance behaviors can vary in different contexts (e.g., red color in a romance-related context may lead to approach behavior, while red in a competition-related context may lead to avoidance behavior)^25^.

Face gender perception is highly silent and socially important for survival, contributing to mate selection and threat detection. It occurs exceptionally quickly in social encounters^26^. Several studies have suggested that sexual dimorphic features of faces, such as face shape cues, serve as a critical foundation for gender perception^27–29^. Walker and Wanke^29^ found that even very subtle facial cues to masculinity and femininity can significantly influence gender categorizations. In real-life situations, faces are typically processed with varying amounts of contextual information, including visual background colors (e.g., clothing color, background color). Recent research has shown that these visual cues can also have important effects on the formation of impressions and perceptions of human faces^30–33^. Cunningham and Macrae^1^ found that a face presented on a background matching its gender (e.g., female face on a pink background, male face on a blue background) elicited faster responses in gender categorization tasks compared to faces presented on gender-mismatching backgrounds (e.g., female face on a blue background, male face on a pink background).

Thus, gender-typed color cues automatically trigger the activation of associated gender representations. However, Cunningham and Macrae^1^ used only two colors (i.e., pink/blue) to test pink–female/blue–male associations, which may have made the hypothesis known to the participants, and it might confound the general saliency effects with the effects of background colors. Most previous studies have examined the effects of facial features or contextual color cues. However, little is known about the effect of the interaction between facial features and background colors on gender perception of faces.

Furthermore, people’s perception of faces is formed based on the interactions of multiple features they have perceived^34^. When the face is inverted, it takes longer to recognize the faces, and also to categorize the gender of the faces^35,36^. Previous studies suggested that face inversion preserves low-level visual features, but disrupts the coding of configural representations of faces, impairing face recognition^37^. More specifically, upright faces are processed holistically, enabling the integration of individual features to maximize the utilization of configural information, whereas inverted faces may be processed more like objects, with relatively independent processing of individual parts^38,39^. If the effect of background color on face gender recognition was independent of the facial configural information, possibly due to the congruency effect of color–gender associations, then the effect of background color should persist even after the inversion manipulation, as the internal facial features are presented in both upright and inverted faces. On the other hand, if the effect of color–gender associations interact with the facial configuration features, leading to a bias in gender categorization of morphed faces, then the effect of background color might differ between the upright and inverted faces. Therefore, examining the effect of background colors in both upright and inverted faces can shed light on the interaction effect between color–gender associations and face configuration information on gender perception of faces, leading to a better understanding of the effect of color on face gender perception.

Based on those previous studies, the current study aimed to fill the research gap by examining the effect of interactions between background color and facial features on gender categorization of human faces. We therefore investigated whether background colors can bias gender categorization of ambiguous sexually dimorphic faces in both upright and inverted conditions. Face stimuli were computer-generated faces whose sexually dimorphic content was morphed monotonically from male to female perception, and presented against three different background colors (i.e., red/green/gray). Previous studies have demonstrated that Japanese participants tend to associate the color red with the perception of female and femininity^6–8,40^. Furthermore, the association between the color red and female may be powerful enough to affect gender-related cognitive processes and behaviors. In a previous study, we found that the automatic activation of the red-female association was evident in Stroop tasks, which facilitated both the categorization of words by gender (male or female) and the discrimination of font colors (green or red)^40^. Thus, red was selected as the color to be associated with female gender among Japanese participants. Green was selected because it is one of the colors associated with male^7,8^ and the opposite color of red in many well-established color models^41^. We did not select blue as a choice due to its prevalence as a more preferred color among both males and females^16,42^, as well as empirical evidence indicating its lack of gender bias^43,44^. Gray was chosen as an achromatic contrasting color that can be matched for saturation and lightness^45,46^. Using two experiments, we first examined whether background colors could interact with the sexual morphic features to bias gender perception of upright faces (Experiment 1). Next, we investigated whether background color influences facial gender categorization independently of facial configuration features. To test this, we examined the interaction between background color and facial inversion on gender perception (Experiment 2). Participants were required to categorize the gender of a face stimulus presented with three different background colors by pressing one of two labelled keys. We hypothesized that the red background color would interact with sexual dimorphic features to bias the gender categorization of faces towards a female perception, and the facial configuration features were also involved in the effect of color on gender categorizations of faces.

## Experiment 1

### Methods

#### Participants

Thirty Japanese undergraduate students (16 males, mean age = 22.1 years, *SD* = 1.8) from Waseda University participated in the experiment. All participants had normal or corrected-to-normal visual acuity and normal color vision, and were naïve regarding the purpose of the experiment. The sample size was set a priori at 23 based on a target of 0.8 power with a medium effect size via power analysis (Cohen’s *d* = 0.6). We collected more data in case some participants’ data could not fit the psychometric functions (e.g., Deviation > 100). This experiment was approved by the institutional review board (IRB) of Waseda University (2015–033), and conducted in accordance with the ethical standards of the 1964 Declaration of Helsinki. Written informed consent was obtained from all individual participants prior to the experiment.

#### Apparatus and stimuli

E-Prime 2.0 (Psychology Software Tools, Inc.) was used to present the stimuli and collect the data. Stimuli were displayed on a 24-inch LCD monitor (EIZO FG2421, EIZO Corp, Hakusan, Japan) with a 1920 × 1080-pixel resolution and a refresh rate of 100 Hz. Participants viewed the monitor binocularly at a distance of approximately 60 cm.

**Figure 1:**
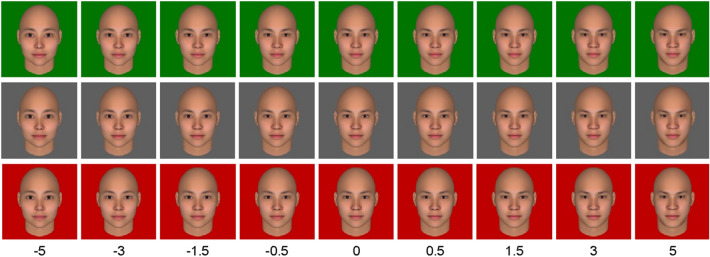
The morphed face stimuli in nine levels of gender standard deviations.

The face stimuli were created using FaceGen Modeller (Singular Inversions, Toronto, Canada). We generated nine versions of an average-looking female face by manipulating sexually dimorphic features from an extremely feminine to an extremely masculine face in a data-driven manner (SD =  − 5, − 3, − 1.5, − 0.5, 0, 0.5, 1.5, 3, 5; Fig. 1). Sexual dimorphic traits of the faces were manipulated based on the data-driven computational model of sexual dimorphism validated in a previous study^47^. Only face shape components were manipulated; remaining facial reflectance components were kept constant. The face stimulus was fitted in a 512 × 512-pixel frame, presented with three different background colors created by Photoshop CS2 (Adobe Systems Inc., San Jose, CA, United States). The three background colors were measured by PR-655 (Photo Research, Chatsworth, CA, USA) and each color was measured 10 times and calculated the average color values. The color information was as follows: Red: L* = 56.66, a* = 80.59, b* = 57.60; Green: L* = 60.03, a* = −64.56, b* = 41.64; Gray: L* = 57.59, a* = −2.46, b* = −30.15.

#### Procedure

**Figure 2:**
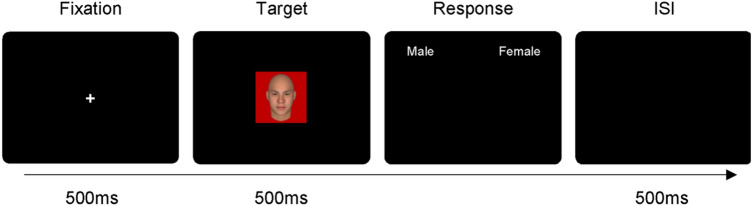
Example of the trial sequence. Participants were required to categorize the gender of the face stimulus (male or female) by pressing one of two labelled keys. ISI = inter-stimulus interval.

The experiment was conducted in a laboratory under dim lighting conditions. At the beginning of the experiment, participants were instructed to categorize the gender of a centrally presented face stimulus by pressing a key on the computer keyboard (e.g., pressing “*z*” for a male face using the left index finger or “*m*” for a female face using the right index finger). Each trial began with a fixation for 500 ms followed by a face stimulus with a background color (i.e., red, blue, or gray) for 500 ms, after which a black screen was presented with two white word cues (male or female) on either side of the screen to remind participants of the correct key assignments until the participants responded (Fig. 2). Participants were asked to categorize the gender of the face stimulus (as male or female) by pressing one of the two labelled keys as quickly as possible. Trials were separated by an inter-stimulus interval of 500 ms. Each face stimulus was presented 12 times in a random order, resulting in 324 trials (9 faces × 3 background colors × 12 repetitions = 324 trials). The experiment was preceded by 20 practice trials. ﻿At the end of every 36 trials, participants took a self-determined break. The entire experiment took approximately 30 min to finish.

#### Analysis

All statistical analyses were performed using R^48^. For each subject and condition of background color (i.e., red, green, and gray), we determined a psychometric function based on the proportion of trials in which the face was judged as female. That is, each face stimulus was repeated for 12 times, the proportion of responses with judging as female was calculated. The rate from each participant was fitted with a psychometric function using a generalized linear model with a binomial distribution. Fitting a psychometric function can reveal the level of stimulus variable at which perceptual performance transitions from one perceptual category to the other (i.e., the point of subjective equality [PSE]) and the precision with which the participant is able to perceptually differentiate stimuli along the dimension (i.e., the just noticeable difference [JND])^49^.

The PSE and JND obtained from the fitted curves were used as indicators of perceived differences in gender judgments and discrimination sensitivity, respectively. The PSE was taken from the *x* value at which the fitted curve had a *y* value of 0.5, i.e., the gender dimorphic value of the face stimuli being judged switched from “female” to “male” (where the face looked equally male and female). Thus, the PSE is the gender dimorphic value denoting the maximum uncertainty of gender information.

The JND was calculated from one-half of the difference between the *x* values at which the fitted curve had *y* values of 0.50 and 0.75. This is the amount of change necessary in a face stimulus for the change to be detected 50% of the time. When the JND value is small, a participant is better able to discriminate the face gender information. The PSEs and JNDs of the three different color backgrounds from the fitted curves of each participant were compared using one-way analysis of variance (ANOVA). A paired-sample *t*-test using Bonferroni’s correction for multiple comparisons (*α* = 0.05/3 = 0.017) was used to further reveal the differences in the PSEs and JNDs between the three background colors. The Bayes Factors (BF10) were further referred to determine whether or not there was support in favor of the alternative (H1) or null (H0) hypotheses^50^. A value of 1 means that null and alternative are equally likely, larger values suggest that the data are in favor of the alternative hypothesis, and smaller values indicate that the data are in favor of the null hypothesis.

To verify the consistency and robustness of the results, the categorical responses were also fitted by means of a Generalized Linear Mixed Model (GLMM)^51^ with the *glmer* function^52^. We included all fixed and random effects as a first instance and then to eliminate those factors that reduced the model’s overall goodness of fit^53^. The Akaike information criterion (AIC) was also used to compare the goodness of fit of the alternative models (a lower AIC value indicating a higher quality model)^54^. After the model selection, analysis of variance (Type II Wald *x*^2^ test) was performed to identify significant effects and interactions, and Tukey post hoc pairwise comparisons were also performed.

The response times (RTs) were analyzed using the linear mixed-effect model analysis by the *lmer* function. We included the background color, face morphing level, gender response, and their interactions as fixed factors in the initial model, and then eliminated factors by evaluating the fitted models using a likelihood ratio test and the AIC values. Analysis of variance (Type II Wald *x*^2^ test) was performed to identify significant effects and interactions. The R code for the data analysis was uploaded in the online supplementary materials.

## Results

RTs that fell 2.5 *SD*s above or below the mean of each participant in each condition of background color were excluded from the analysis (3.28% of all trials). Five participants whose responses failed to fit the psychometric function were removed from data analysis (see plots of psychometric functions of gender categorization for varying levels of face morphing in each of the participants in Supplementary materials). Thus, 25 participants’ data were used for further data analysis. We calculated the overall male and female responses from each participant, irrespective of the extent of facial gender manipulation, and found that participants made male responses more frequently than female responses, *t*(24) = 5.14, *p* < 0.001, Cohen’s *d* = 1.03.

**Figure 3:**
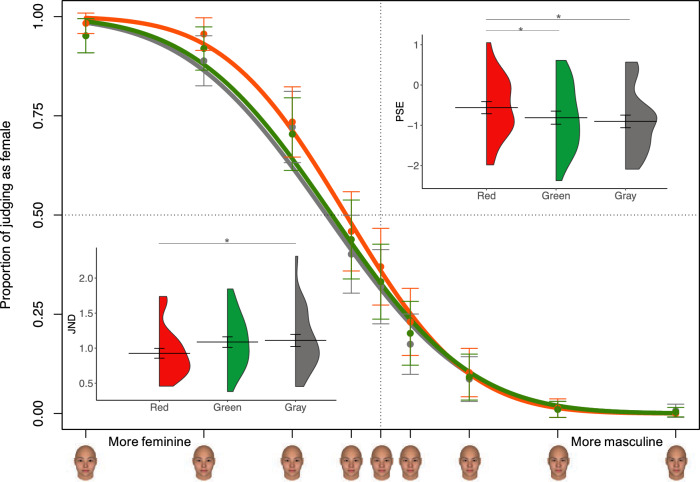
The proportion of female responses for each face stimulus by psychometric functions. The horizontal axis shows the face morphing level. The vertical axis indicates the proportion of male responses. Colored curves show the logistic fits to the psychometric results for the three background colors. The violin plot showing the distribution of individual PSEs and JNDs in the three background colors are presented. The horizontal line represents the mean. Error bars represent the standard mean error. Asterisks indicate significant differences (*p < .05, Bonferroni corrected).

The mean proportion of female responses for each face stimulus and their psychometric curves with the three background colors are shown in Fig. 3. The data points represent the mean proportion of trials in which participants responded with female judgement for each morphed face stimulus presented with three background colors. The PSE would be larger than zero if the face was perceived as more feminine than masculine and smaller than zero otherwise. Thus, the face stimuli tended to be perceived as more masculine than feminine with the three PSEs smaller than zero (Fig. 3), consistent with the result that participants made more male responses. A one-way ANOVA of the PSE showed a significant main effect of background color (Fig. 3), *F*(2, 48) = 5.68, *p* = 0.006, η_p_^2^ = 0.03, BF10 = 6.72. The PSEs were significantly larger for the red background color (Mean =  − 0.56, SD = 0.75) than for the green (Mean =  − 0.81, SD = 0.81; *t*[24] = 2.88, Bonferroni corrected *p* = 0.02, Cohen’s *d* = 0.58, BF10 = 5.65) and gray background colors (Mean =  − 0.90, SD = 0.79; *t*[24] = 2.76, Bonferroni corrected *p* = 0.03, Cohen’s *d* = 0.55, BF10 = 4.48). No difference in PSEs was observed between the gray and green background colors *t*[24] = 0.91, Bonferroni corrected *p* = 1, Cohen’s *d* = 0.18, BF10 = 0.31). Thus, the red background color biased the gender categorization of faces toward a female face perception, and reduced the shift toward a male face perception compared with green and gray background colors.

A one-way ANOVA of JNDs showed a significant difference among the three background colors (Fig. 3), *F*(2, 48) = 5.07, *p* = 0.01, η_p_^2^ = 0.04, BF10 = 4.51, suggesting that the sensitivity of face gender perception could be influenced by background colors. A paired-sample *t*-test showed that JNDs were significantly smaller for the red background color (Mean = 0.93, SD = 0.35) than for gray background colors (Mean = 1.11, SD = 0.43; *t*[24] = 2.85, Bonferroni corrected *p* = 0.027, Cohen’s *d* = 0.57, BF10 = 5.29), and a marginal difference from the green background color (Mean = 1.09, SD = 0.38; *t*[24] = 2.53, Bonferroni corrected *p* = 0.054, Cohen’s *d* = 0.51, BF10 = 2.90). No difference was observed between the gray and green background colors *t*[24] = 0.38, Bonferroni corrected *p* = 1, Cohen’s *d* = 0.08, BF10 = 0.22).

As a further test of population categorical responses, these were fitted using a GLMM. We anticipated that the gender perception of faces would depend on those two factors with the background color and the face morphing levels. The generalized linear mixed-effect model analysis showed that there was no significant interaction effect between the background color and face morphing levels, *x*(16)^2^ = 10.31, *p* = 0.85. After removing the interaction effect from model analysis, a final model including the background color and face morphing levels as fixed factors (AIC = 5252.99) showed a significant main effect of background color, *x*(2)^2^ = 13.07, *p* = 0.001, and face morphing levels *x*(8)^2^ = 1862.96, *p* < 0.001. Post-hoc multiple comparison showed a significant difference on the logit scale between the red and gray background colors (Tukey’s HSD, *p* = 0.001), and between the red and green background colors (Tukey’s HSD, *p* = 0.04). No difference was observed between the gray and green background colors (Tukey’s HSD, *p* = 0.50). These analyses performed on the categorical responses led to the same conclusion: the red background color was more likely to bias face stimulus towards a feminine face than green and gray background colors.

### Response times

The linear mixed-effect model analysis showed that there was no significant interaction effect between background color, face morphing levels, and gender response on the RTs, *x*(15)^2^ = 10.48, *p* = 0.79, or interactions between background color and face morphing levels, *x*(16)^2^ = 6.41, *p* = 0.98, or between background color and gender responses, *x*(2)^2^ = 3.72, *p* = 0.16. There was no significant main effect of background color on RTs, *x*(2)^2^ = 1.06, *p* = 0.59. Those results suggested that the background color played little effect on RTs for categorizing gender of faces.

In summary, participants tended to make more female responses in gender judgment of faces, when the faces are presented in a red background color, compared with green and gray background colors. This suggests a red effect biasing face-gender toward female face perception. Moreover, participants found it easier (lower JNDs) to make a gender judgment when it was presented on a red background color than gray. These results may indicate a congruency effect of red–female associations on the gender categorization of faces. However, it is unknown whether this effect of red background color was independent from the facial configural features. In the next experiment, we used inverted face stimulus, to further reveal the interaction effect between red background color and facial configural features on face-gender judgments.

## Experiment 2

Experiment 1 demonstrated that a red background color interacting with sexual dimorphic features biases the gender categorization of an ambiguous upright face toward a female face compared with green and gray background colors. In Experiment 2, we manipulated the face inversion to test whether the effect of red background color on face gender recognition was independent of the facial configural information. Previous studies suggested that face inversion disrupts the facial configuration and relationships between facial features, making it difficult for the brain to accurately identify gender based on facial features^55,56^. If the observed effect of red background color is independent of facial configuration, a similar effect of red-female association is expected in the gender categorization of inverted faces. On the other hand, if facial configuration is necessary for the effect of red-female association in biasing face perception, there will be limited effect of red background color in the categorization of gender for inverted faces. Thus, manipulating face inversion would be helpful to further reveal the interaction effect between red background color and facial configural information on gender judgment of faces. Furthermore, the ability to recognize faces is crucial for social interactions. When faces are inverted, it can impede the understanding of facial expressions and emotions. Meanwhile, color-gender associations are learned from social interactions that involve co-occurrences of gender categorization and colors. Therefore, studying the impact of color-gender associations on inverted faces can aid in comprehending the relationship between color and face recognition learned from social interactions.

### Methods

#### Participants

Twenty-five newly recruited Japanese undergraduate students (14 male, mean age = 20.3 years, *SD* = 1.7) from Waseda University participated in the experiment. All participants had normal or corrected-to-normal visual acuity and normal color vision. All participants gave their informed written consent before participating in the study, which was approved by the institutional review board (IRB) of Waseda University (2015-033), and conducted in accordance with the ethical standards of the 1964 Declaration of Helsinki.

#### Stimuli and procedure

The face stimuli were identical to those used in Experiment 1 except that they were inverted. The experimental setting and procedure were identical to those of Experiment 1.

#### Analysis

The data analysis was identical to that in Experiment 1.

## Results

RTs that fell 2.5 *SD*s above or below the mean of each participant in each condition of background color were excluded from the analysis (3.64% of all trials). Two participants whose responses failed to fit the psychometric function were removed from the data analysis (see plots of psychometric functions from each of participants in Supplementary materials). Thus, 23 participants’ data were used for data analysis. For the total amount of male and female responses, irrespective the extent of facial gender manipulation, participants made male responses more frequently than female responses, *t*(22) = 2.80, *p* = 0.01, Cohen’s *d* = 0.58, consistent with Experiment 1.

**Figure 4:**
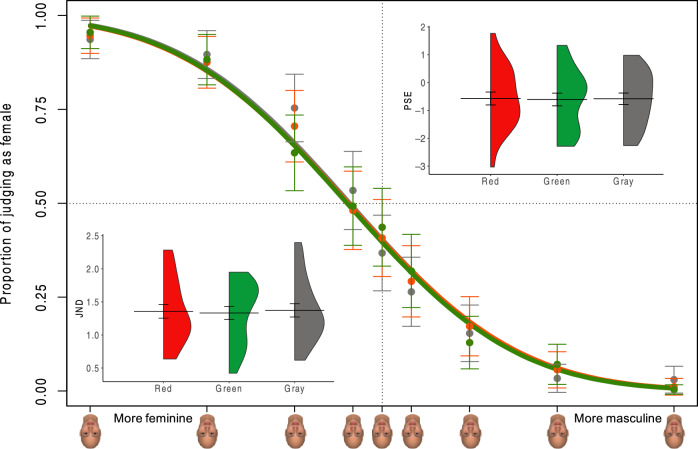
The proportion of female responses for each inverted face stimulus by psychometric functions. Colored curves show the logistic fits to the psychometric results for the three background colors. The violin plot showing the distribution of individual PSEs and JNDs in the three background colors are presented. The horizontal line represents the mean. Error bars represent the standard mean error.

The proportion of female responses to each inverted face stimulus and their psychometric curves with the three background colors are shown in Fig. 4. A one-way ANOVA of the PSE showed no significant main effect of the background colors (Fig. 4), *F*(2, 44) = 0.04, *p* = 0.96, η_p_^2^ = 0.00, BF10 = 0.12. Thus, there were no differences in gender categorization of inverted faces with the red, green, or gray background colors. One-way ANOVA of JNDs showed no significant differences among the three background colors (Fig. 4), *F*(2, 44) = 0.07, *p* = 0.93, η_p_^2^ = 0.00, BF10 = 0.13, suggesting that the sensitivity of gender perception for inverted faces could not be influenced by the background colors.

A final model including the interaction and main fixed factors (AIC = 5714.93) showed a significant interaction effect between the background color and face morphing levels, *x*(16)^2^ = 30.38, *p* = 0.02, a significant main effect of face morphing levels, *x*(8)^2^ = 1739.37, *p* < 0.001, and no significant main effect of background color, *x*(2)^2^ = 0.19, *p* = 0.91. Thus, the background color showed little effect on gender categorization of inverted faces.

### Response times

The linear mixed-effect model analysis showed that there was a significant interaction effect between background color, face morphing levels, and gender response on the RTs, *x*(16)^2^ = 28.67, *p* = 0.03, and no significant interactions between background color and face morphing levels, *x*(16)^2^ = 17.47, *p* = 0.36, and no significant interaction effect between background color and gender responses, *x*(2)^2^ = 3.76, *p* = 0.15. There was no significant main effect of background color on RTs, *x*(2)^2^ = 0.06, *p* = 0.97. Thus, the background color played little effect on RTs for gender categorizing of inverted faces.

In summary, all the analyses performed on the categorical responses and response times showed no significant effect of background color on gender categorization of inverted faces. The effect of red background color on biasing towards a female face observed in Experiment 1 was diminished when the face stimulus was presented in inversion. As face inversion disrupted the face configuration^37^, those results suggest that facial configural features are involved in the effect of a red background biasing toward a perception of a female face.

## Discussion

The present study examined the effect of interaction between background colors and facial features on gender categorization of upright (Exp. 1) and inverted (Exp. 2) faces. Results showed that a red background color could bias gender perception of an ambiguous upright-face stimulus toward a female face compared with green and gray background colors. However, no significant effect of background color was observed on gender perception of inverted faces. These results suggest that red background color interacted with facial configural features to bias the gender processing of faces toward a female face perception.

When used as a background color to present an upright face stimulus whose sexual dimorphic content was manipulated, red background color could automatically activate the learned red–female associations, interrupting the gender processing, and lead to a female face bias, compared with green and gray background colors. These results were consistent with previous findings, suggesting a congruent effect of color–gender associations on processing of gender-related semantics and objects^1,3,6–8,20,40,57^. For instance, Cunningham and Macrae^1^ reported that pink and blue color cues prompted the automatic activation of gender-related categorical representations during the forename-classification task and sex-typed objects (e.g., football or bras) task.

However, when the face stimulus was inverted, there was no effect of background color on gender categorization, and the interaction effect of red background color and sexual dimorphic features was diminished. Previous studies have suggested that face inversion disrupts holistic processing of facial configural features^38,55^. This impairment of high-level facial features, such as relational information about the distance between the nose and mouth, substantially impairs face-gender categorization^39,55,56^. The limited effect of color-gender associations on face gender perception may be influenced by facial configural information. Specifically, the effect of a red background color on biasing face gender toward a female face seems to depend on the representation of facial configuration. Thus, when face configuration information was disrupted, the effect of red-female association observed in Experiment 1 was diminished. These results support the idea that red-female associations are learned and require interaction with high-level facial configural information to have a congruency effect on gender categorization of ambiguous faces^40^. Furthermore, inverted faces are generally more difficult to recognize and process compared to upright faces, due to the disruption of the facial configuration^35,36^. Our participants tended to respond slower in Experiment 2 (mean RT = 435.59 ms, *sd* = 466.52) than those in Experiment 1 (mean RT = 390.67 ms, *sd* = 425.80), consistent with previous findings. The difficulty in extracting configural information from inverted faces may impede the activation of associations between the color red and the female gender in gender categorization. Future studies are in need to further examine the direct interaction effect between face recognition and color-gender associations.

Notably, green-male association showed little effect on the gender categorization of faces. This suggests that the association between green and male may not be strong. Another possibility is that gray was used as an achromatic contrasting color, which could also be associated with male. Previous studies have shown that black may be associated with masculinity, and that toilet pictograms in black are recognized faster when distinguishing male concepts from female concepts^7^. Future studies are needed to examine the colors associated with masculinity and to test the strength of those associations.

A growing number of studies have shown that colors are associated with gender information (e.g., pink–female, blue–male)^1–3^. The early perception of gender-stereotyped colors in infants and children and frequently encountered co-occurrences of reddish colors and female images/concepts (e.g., rouge blush in cosmetics, red clothing in female fashion, red symbols female in public service) in later adults may provide a Bayesian basis for forming red–female associations, which are enhanced through statistical learning and language development in social interactions. In visual arts and literature, red has also long been associated with feminine characteristics (e.g., attractiveness; *The Scarlet Letter*)^46,58–60^. A large number of studies reported an implicit association between red and attractiveness on romance related information processing and behaviors^24,46^. Moreover, girls and women showed affective preference for reddish colors while boys and men tended to prefer blue/green colors more^11,12^. The gender differences in color preference may also suggest an emotion basis for color–gender associations.

Previous studies suggested that top-down factors (e.g., stored knowledge, emotion, contexts) of colors influence the face perception and cognition^61–63^. Color perception automatically activates an associated semantic/emotion representation (e.g., seeing yellow primes banana-like shape)^45,64–66^ and influence the low-level perceptual processing. For instance, the Stroop effect demonstrates that recognition of feminine word could be facilitated when the font color is red, and discrimination of red font color (compared with green font color in different saturation levels) could be facilitated when the word meaning is feminine^40^. The current study may suggest a top-down effect of learned red–female associations interacting with facial configural cues to play a congruency effect on face gender categorization.

It should be noted that red has also been associated with dominance, aggression, and anger, which are related to masculine characteristics^33,45,67,68^. Young et al^45^ reported that red background color could facilitate anger face classification. Chen et al^69^ observed that red background color enhanced a dominant face perception compared with control colors. Given that facial anger/dominance and masculinity is positively correlational (the more masculine the face is, the more dominant/angry it looks)^70^, the present finding that red background color bias toward a female gender perception is an opposite effect. According to the color-in-context theory^23,24^, color conveys psycho-functional information that is influenced by the context, and it could be either biologically based, based on learned associations, or both. The present study suggests that red–female associations are learned high-level associations more than biologically exhibited. The effect of a red background color bias towards a female face perception was contributed to by the interaction between high-level red-female associations and high-level facial configuration representations. Future works are in need to further reveal the mechanism of red–gender associations and clarify the effect of statistical learning on these associations. It could be interesting to examine individual differences on color–gender associations, such as testing the development of those associations (e.g., whether children show color–gender associations), and the effect of cultural differences on these association. For instance, a famous Chinese four-character proverb—“red–men green–women” suggest an opposite color–gender association as gender–color stereotypes in modern western culture. In China, red have positive meanings different from western cultures, such as representing happiness, good luck, fortune, power, and influences^71,72^. Learning individual/developmental/cultural differences may give a better understanding on the nature of color–gender associations.

One limitation of the present study is that we used face stimuli of computer-generated morphed faces, which were hairless. This might partially explain why our participants made male responses more frequently than female responses. It might also have influenced the effect of red background color on gender perception of faces. Future studies can use morphed real face stimuli to explore the effect of background color on gender perception. Moreover, the interaction effect between facial features (e.g., skin texture) and red color (e.g., background color/skin color/clothing color) might be interesting for future studies, for instance, by fitting the psychometric function to reveal the effect of red facial color on gender categorization. The current study's sample size may not have been sufficient to reveal any gender differences among perceivers in face processing. Previous studies have reported that females are better at recognizing female faces and other basic face perceptions such as face emotions^73,74^. Therefore, it is possible that gender differences exist in the observed effect of red background color on gender categorization of faces. Future studies with larger sample sizes could examine the interaction between perceivers' gender and the effect of red-female associations on face gender perception.

In summary, the current study revealed an effect of interaction between red background color and facial configuration information on bias gender categorization of faces toward a female face perception. These results suggest that learned high-level red–female associations modulate the face-gender perception toward a female face, perhaps through a top–down processing. The findings of this study add to a growing body of evidence for the effect of red on face gender perception, pointing to a red–female association. Future studies are needed to examine the formation of color–gender associations and to reveal the mechanism underlying those associations.

## Data Availability

The raw data and R code for this article are available online: https://osf.io/2uj6b/.
